# Subpathway-CorSP: Identification of metabolic subpathways via integrating expression correlations and topological features between metabolites and genes of interest within pathways

**DOI:** 10.1038/srep33262

**Published:** 2016-09-14

**Authors:** Chenchen Feng, Jian Zhang, Xuecang Li, Bo Ai, Junwei Han, Qiuyu Wang, Taiming Wei, Yong Xu, Meng Li, Shang Li, Chao Song, Chunquan Li

**Affiliations:** 1 School of Medical Informatics, Daqing Campus, Harbin Medical University, Daqing 163319, China; 2 College of Bioinformatics Science and Technology, Harbin Medical University, Harbin 150081,China; 3 School of Nursing, Daqing Campus, Harbin Medical University, Daqing 163319, China; 4 School of Pharmacy, Daqing Campus, Harbin Medical University, Daqing 163319, China; 5 The fifth Affiliated Hospital of Harbin Medical University, Daqing 163319, China; 6 Department of Pharmacology, Daqing Campus, Harbin Medical University, Daqing 163319, China

## Abstract

Metabolic pathway analysis is a popular strategy for comprehensively researching metabolites and genes of interest associated with specific diseases. However, the traditional pathway identification methods do not accurately consider the combined effect of these interesting molecules and neglects expression correlations or topological features embedded in the pathways. In this study, we propose a powerful method, Subpathway-CorSP, for identifying metabolic subpathway regions. This method improved on original pathway identification methods by using a subpathway identification strategy and emphasizing expression correlations between metabolites and genes of interest based on topological features within the metabolic pathways. We analyzed a prostate cancer data set and its metastatic sub-group data set with detailed comparison of Subpathway-CorSP with four traditional pathway identification methods. Subpathway-CorSP was able to identify multiple subpathway regions whose entire corresponding pathways were not detected by traditional pathway identification methods. Further evidences indicated that Subpathway-CorSP provided a robust and efficient way of reliably recalling cancer-related subpathways and locating novel subpathways by the combined effect of metabolites and genes. This was a novel subpathway strategy based on systematically considering expression correlations and topological features between metabolites and genes of interest within given pathways.

Metabolic dysfunction is a primary cause of many complex diseases that have become prevalent in humans such as prostate cancer, breast cancer and cardiovascular disease^1^,^2^,^3^. In recent years, various ‘omics’ technologies including genomic, transcriptomics, proteomics and metabolomics have grown. These technologies are concerned with comprehensive analysis of metabolites and genes associated with specific diseases. Metabolic pathway analysis has become a successful strategy for understanding large amounts of molecules generated by these ‘omics’ technologies. Methods such as overrepresentation approach and gene set enrichment analysis have been developed and widely applied to identify pathways^4^,^5^,^6^,^7^,^8^,^9^,^10^,^11^,^12^,^13^. However, most of these methods use gene information only and not the combined effect of metabolite and gene analysis^4^,^10^,^11^,^12^,^13^,^14^,^15^. Because metabolic pathways involve both metabolites and genes, their dysfunction is closely related to pathogenesis of complex diseases^16^,^17^,^18^. Thus, integrative pathway analysis of both metabolites and genes would help in interpreting metabolic function and its underlying biological significance. Recently, a metabolic pathway analysis method, IMPaLA, was proposed by Kamburov *et al*. to identify pathways^16^. This method integrated the enrichment significance of pathways based on overrepresentation approach, which was calculated by multiplying the P-values of metabolites and genes, to improve pathway identification performance. However, IMPaLA did not use expression correlations or topology embedded in the pathways, regarding pathways as simple gene sets. Metabolites or genes in a pathway do not always show the same topological importance. Therefore, simply considering the statistical features might not comprehensively reflect the superiority of pathway analysis. Moreover, metabolic pathways are often too large to interpret the relevant biological phenomena accurately. Key subpathway regions of the entire corresponding pathway may be more useful for reflecting the relevant biological importance^19^. Many studies have confirmed that abnormal subpathway regions of metabolic pathways were likely to result in human complex diseases^15^,^20^,^21^.

In our previous study, we developed an analytical method called Subpathway-GM to identify biologically meaningful metabolic subpathways. We analyzed lenient distance similarities of important nodes within metabolic pathways to locate key subpathway regions^19^. The results demonstrated that subpathway identification strategy along with pathway topology was able to identify disease-related subpathway regions successfully and reliably. However, Subpathway-GM did not fully consider expression correlation information of metabolites and genes within the pathways. Metabolic pathways are the classical focus in systems biology for studying the dynamic changes of metabolites and genes in a given biological context^22^. In metabolic pathways, genes and families of enzymes control cellular metabolic phenotypes through the totality of small-molecule metabolites. The biological variation of metabolite concentrations reflects tiny homeostatic adjustments that maintain the metabolome in a steady state^23^. In chemical reactions, enzymes catalyze reactions at different efficiencies and rates. The chemical processes controlled by enzymes involve metabolites as substrates, intermediates or end-products. Important concentration changes of some enzymes can lead to important changes in metabolite concentrations^24^. In addition, metabolite concentration changes in cells could also be controlled by intrinsic genetic alterations. Perturbations of metabolite concentrations indirectly affect the expression of genes encoding enzymes, and expression changes of these genes reflect the topology of the pathway^25^. In summary, the molecules within the metabolic pathways, including metabolites and genes, influence each other through expression levels or molecular concentrations. The demonstration of the expression correlations in biological systems has opened a new window on metabolic research, allowing monitoring of the effects of cellular perturbations on metabolic pathways^26^. Therefore, even though specifying the differential expression of metabolites or genes is currently possible, discussing the correlation between expression changes in genes encoding enzymes and changes in metabolite concentrations is pivotal for a more comprehensive picture of metabolic pathways^27^. In conclusion, we propose that expression correlations of metabolites and genes should be considered to locate key metabolic subpathway regions related to the underlying biological phenomena of complex human diseases. Knowing these correlations would provide significantly more information about metabolic system.

In this study, we proposed a powerful method called Subpathway-CorSP to identify metabolic subpathway regions by integrating expression correlations and topological features of metabolites and genes of interest within pathways. Importantly, we improved on original pathway identification methods by using subpathway identification strategy and emphasizing expression correlations between metabolites and genes of interest based on topological features within the metabolic pathways. First, we integrated differentially expressed (DE) metabolites, DE genes, as well as metabolites and genes with high expression correlation related to the study condition (prostate cancer) into the corresponding compound and enzyme nodes (nodes of interest) within reconstructed metabolic pathway graphs. Then, we considered expression correlations between metabolites and genes of interest based on topological features to locate key metabolic subpathway regions whose internal structures were highly correlated for expression. Finally, we used hypergeometric test to evaluate the enrichment significance of these subpathway regions. Subpathway-CorSP was applied to a prostate cancer data set and its metastatic sub-group data set. We demonstrated that the method was able to identify multiple disease-related subpathway regions robustly and efficiently.

## Results

**Figure 1:**
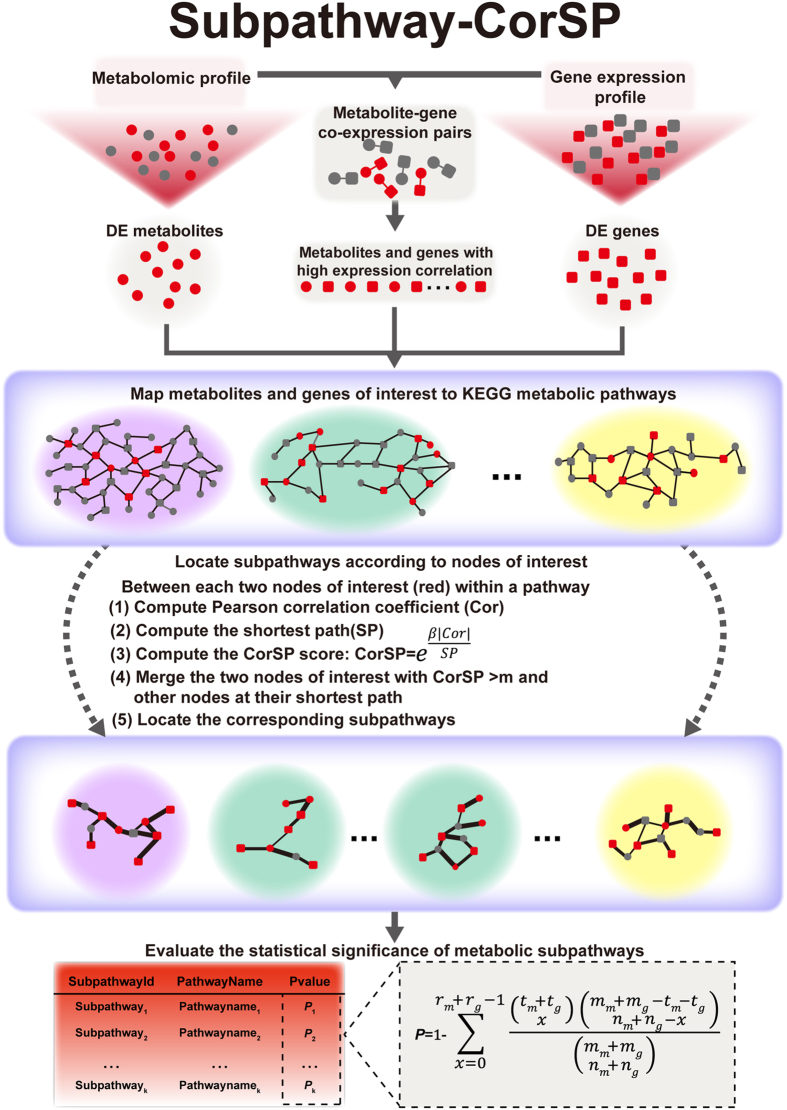
Schematic overview of Subpathway-CorSP. (i) Map metabolites and genes of interest to graphs of metabolic pathways after graph-based reconstruction of metabolic pathways. (ii) Develop subpathway identification algorithm and locate metabolic subpathways within pathways according to nodes of interest. (iii) Evaluate the statistical significance of metabolic subpathway regions.

We applied Subpathway-CorSP to the identification of prostate cancer-related metabolic subpathways. The schematic overview of Subpathway-CorSP is in Fig. 1. To demonstrate that Subpathway-CorSP would identify meaningful metabolic subpathways, we compared Subpathway-CorSP with other methods at the system level. Four methods were used to identify pathways for the same data set: Pathway-G, Pathway-M, IMPaLA and Subpathway-GM. These methods are commonly used for pathway identification and have been included in many pathway analysis tools^4^,^8^,^9^,^16^. Pathway-G uses only DE genes to identify entire pathways via hypergeometric test^4^. Similar to Pathway-G, Pathway-M uses only DE metabolites^7^,^8^,^9^. IMPaLA integrates DE metabolites and DE genes to identify entire pathways via hypergeometric test, without considering pathway structure or a subpathway identification strategy^16^. Subpathway-GM integrated DE metabolites and genes to identify metabolic subpathways, considering their positions and cascade regions within the given pathway, but without expression correlation information^19^.

### Subpathway-CorSP identified meaningful metabolic subpathways

We identified 48 DE metabolites by Wilcoxon rank-sum test (P < 0.05) and 1182 DE genes by SAM method (FDR < 0.001) between benign prostate samples (Benign) and prostate cancer samples (PCA+ Mets). Meanwhile, we identified 40 metabolites and 5581 genes with high expression correlation using Pearson correlation coefficient (Pcc) >0.8. We obtained 63 metabolites and 5846 genes of interest by combining a union set of the above metabolites and genes (Table 1). With these nodes of interest, Subpathway-CorSP located 92 potential metabolic subpathways with s = 5 from all metabolic pathways (Supplementary Data set S1). We set the parameter s = 5, similar to the previous Subpathway-GM method^19^. This type of subpathway (s ≥ 5) has been reported to be associated with cancer in some studies and is considered to represent a pathway^19^. In contrast, a small-scale node set (s < 5) was a scatter node set lacking sufficiently biological significance.

**Figure 2:**
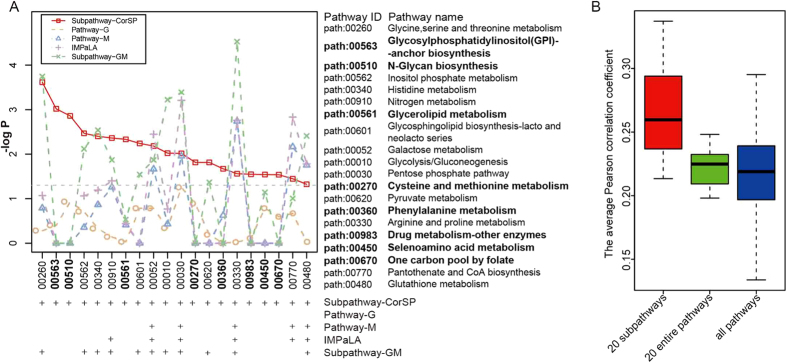
Identification of metabolic subpathways associated with prostate cancer. (**A**) Plots of pathway significance (–log10 P-value) in Subpathway-CorSP, Pathway-G, Pathway-M, IMPaLA and Subpathway-GM. Subpathway-CorSP identified 20 significant metabolic subpathways, corresponding to 20 entire pathways. Plus sign, pathway was identified by the corresponding method at the 5% significance level. Bold labels, additional pathways identified only by Subpathway-CorSP. (**B**) Boxplot of average Pcc value for the 20 significant subpathways, the corresponding 20 entire pathways and all metabolic pathways. Average Pcc value for the 20 significant subpathways was significantly higher than for the corresponding 20 entire pathways (P = 2.861e-06) and significantly higher than for all metabolic pathways (P = 1.271e-05).

With a statistical significance level of 0.05, Subpathway-CorSP identified 20 significant metabolic subpathways. These subpathways also corresponded to 20 entire metabolic pathways (Fig. 2A). Of these pathways, 18 (90%) were well reported to be associated with cancer (Table 2). Many pathways identified by Subpathway-CorSP were undetected by Pathway-G, Pathway-M, IMPaLA or Subpathway-GM. Pathway-G located 62 entire metabolic pathways, but did not find any significant pathways at the 5% significance level (Supplementary Data set S1). Thus, all the significant pathways identified by Subpathway-CorSP were not identified by Pathway-G (Fig. 2A). Pathway-M located 42 potential metabolic pathways and identified 13 significant pathways at the 5% significance level (Supplementary Data set S1). However, Subpathway-CorSP identified an additional 15 (75%) not identified by Pathway-M (Fig. 2A). The limited abilities could also be seen in IMPaLA and Subpathway-GM. IMPaLA found 15 significant pathways (P < 0.05) from 32 potential metabolic pathways (Supplementary Data set S1). However, 70% of the 20 significant pathways identified by Subpathway-CorSP were not considered significant by IMPaLA (Fig. 2A). Subpathway-GM identified 29 significant subpathways with a cut-off value of P < 0.05 (Supplementary Data set S1). These subpathways corresponded to 28 entire pathways, 13 of which were identified by Subpathway-CorSP. Of the 20 significant pathways identified by Subpathway-CorSP, 45% were not considered significant by Subpathway-GM (Fig. 2A).

Subpathway-CorSP tended to locate key subpathway regions that mainly considered expression correlation information. Therefore, we tested if the significant subpathways identified showed clearly higher expression correlations. We obtained all Pcc values of metabolite/gene pairs within 20 significant subpathways and calculated those within any single metabolic pathway. The average Pcc value for the 20 significant subpathways was significantly higher than for the corresponding 20 entire pathways (P = 2.861e-06, Wilcoxon rank-sum test) (Fig. 2B). The average Pcc value for the 20 significant subpathways was also significantly higher than for all the metabolic pathways (P = 1.271e-05, Wilcoxon rank-sum test) (Fig. 2B). These results demonstrated that Subpathway-CorSP improved subpathway identification by emphasizing expression correlations between metabolites and genes of interest.

We noticed that up to eight pathways identified by Subpathway-CorSP were simultaneously not detected by Pathway-G, Pathway-M, IMPaLA and Subpathway-GM (Table 2). These pathways either had no significant P-values or were not found by Pathway-G, Pathway-M, IMPaLA and Subpathway-GM. We focused on three of the eight additional pathways that contained both metabolites and genes of interest.

**Figure 3:**
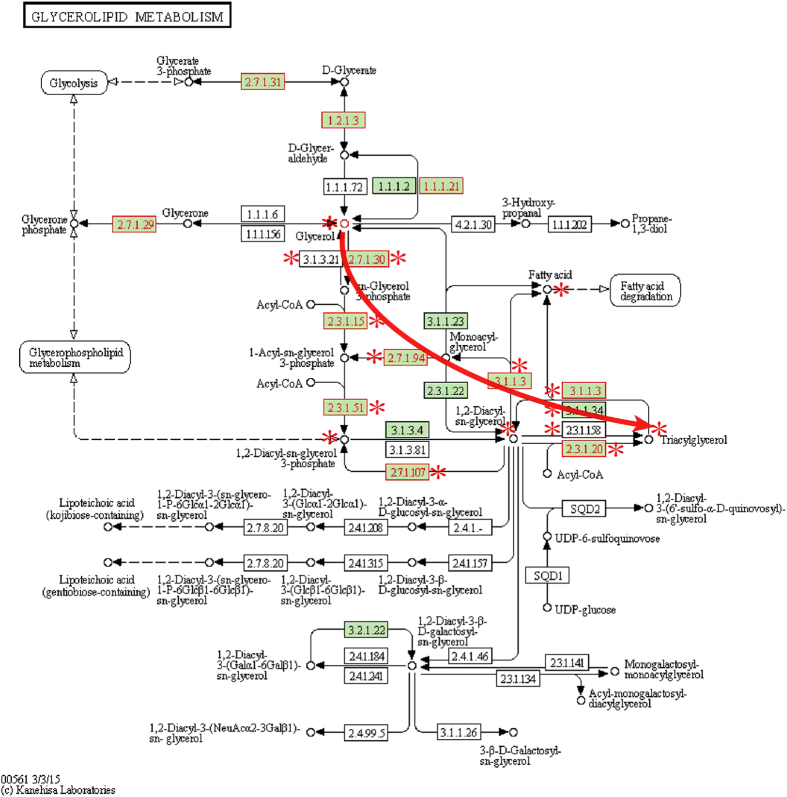
Glycerolipid metabolism pathway with metabolites and genes of interest annotated. Nodes near *symbol, key metabolism subpathway region (path:00561_1) identified by Subpathway-CorSP. Red node labels and borders, enzymes (rectangular nodes) mapped by genes of interest. Red node borders, metabolites (circle nodes) mapped by metabolites of interest.

**Figure 4:**
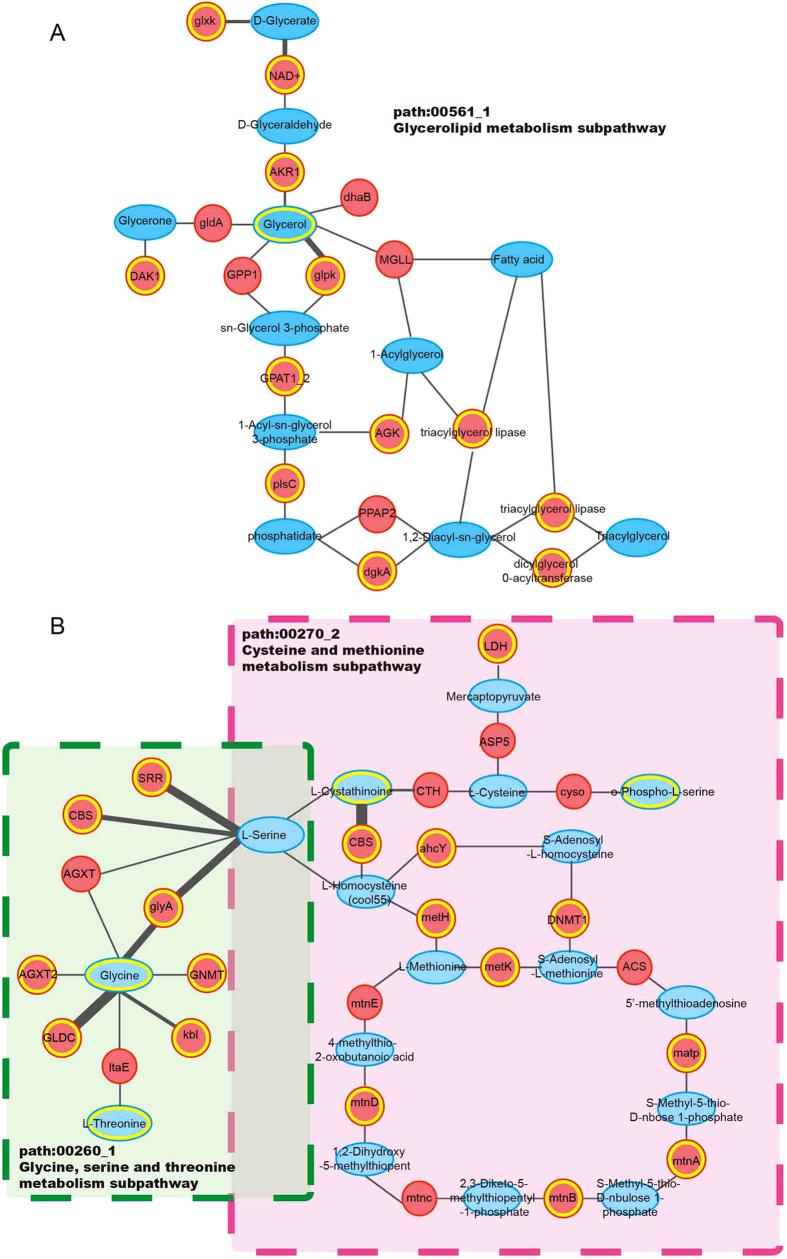
Significant subpathway regions identified by Subpathway-CorSP. (**A**) Plot of key glycerolipid metabolism subpathway (path:00561_1, P = 0.00464). (**B**) Plot of key cysteine and methionine metabolism subpathway (path:00270_2, P = 0.0153). This subpathway had a common metabolite (serine) with glycine, serine and threonine metabolism subpathway, which was the most significant metabolic subpathway (path:00260_1, P = 0.00024). Blue ellipse, metabolites; red circle, genes. Nodes with yellow edge, metabolites and genes of interest in the pathway.

The most significant subpathway (path:00561_1) of the three additional subpathways was in glycerolipid metabolism pathway (Fig. 3). Subpathway-CorSP yielded a P-value of 0.00464, but was not considered significant by Pathway-G, Pathway-M, IMPaLA or Subpathway-GM (P > 0.1). Glycerolipids usually refer to esters generated by esterification of glycerols and fatty acids, including saturated fatty acids and unsaturated fatty acids, which are important in killing tumor cells, inducing cell apoptosis and enhancing cellular and humoral immunity^28^. Thirty years ago, glycerolipids were investigated biochemically as novel cellular signaling entities. These biomolecules occupy signaling nodes critical to a number of physiological and pathological processes^29^. Glycerol-based lipids have prominent functions in human physiology and complex diseases from fat storage and metabolic disorders to survival pathways in cancers^30^. Moreover, the key region (Fig. 4A) where glycerol is converted to triacylglycerol by glycerol kinase, acyltransferase, acylglycerol kinase and other enzymes is closely related to cellular proliferation, carcinogenesis and cancer survival and mortality risk^31^. Lipases in this subpathway region effectively affect prostate cancer cell survival and invasion *in vitro* and reduce prostate tumor growth^32^. Compound and enzyme nodes mapped by metabolites and genes of interest were closely located in the core subpathway region of the glycerolipid metabolism pathway. The average Pcc value for the glycerolipid metabolism subpathway was significantly higher than for the corresponding entire pathway (Supplementary Table S1). This result indicated that high expression correlations could help locate important subpathway regions and explain biological phenomena.

The second significant subpathway (path: 00270_2) belonged to a cysteine and methionine metabolism pathway and had a P-value of 0.0153 (Fig. 4B). The corresponding pathway was not considered significant by Pathway-G (P = 0.129), and was not found by Pathway-M, IMPaLA and Subpathway-GM. In this pathway, methionine metabolites, including cystathionine and cysteine, significantly increased the ability to predict aggressive prostate cancer^33^. Furthermore, methionine metabolism involves mechanisms for sarcosine formation, and sarcosine is a potential key metabolic intermediary of prostate cancer cell invasion and aggressivity^18^. Cystathionine is at the center of the cysteine and methionine metabolism pathway. From a biological point of view, the center of the pathway where nodes of interest locate displays important information. In addition to cystathionine, closely connected enzymes encoded by genes of interest include adenosylhomocysteinase, methyltransferase and methylthioadenosine phosphorylase, which form an approximately circular subpathway region. This subpathway had significantly higher average Pcc value than the corresponding entire pathway (Supplementary Table S1), demonstrating the importance of expression correlations in subpathway identification. Serine, which is converted by galactitol to cystathionine, is also a core metabolite in glycine, serine and threonine metabolism (Fig. 4B). Glycine, serine and threonine metabolism was the most significant metabolic subpathway (a P-value of 0.00024) identified by Subpathway-CorSP and is reported to be highly associated with metastatic prostate cancer^18^. In conclusion, Subpathway-CorSP identified prostate cancer-related subpathways and tended to locate key regions effectively, using both expression correlations and topological features between nodes of interest within the metabolic pathways.

The third significant subpathway (path:00360_1) belonged to a phenylalanine metabolism pathway (Supplementary Figure S1). Subpathway-CorSP analysis yielded a P-value of 0.0213 for this subpathway. The corresponding pathway was not considered significant by Pathway-M (P = 0.238), and was not found by Pathway-G, IMPaLA and Subpathway-GM. This subpathway was not at the core region of the corresponding pathway, but is reported to be highly associated with cancer (Table 2). Two metabolites of interest in this subpathway, phenylalanine and tyrosine, are novel potential biomarkers for bladder cancer. These potential biomarkers have diagnostic value and indicate the risk of cancer recurrence^34^. Other research shows that selective tyrosine and phenylalanine restriction target mitochondria to induce apoptosis of DU145 and PC3 prostate cancer cells^35^. An enzyme encoded by a gene of interest, aromatic-L-amino-acid decarboxylase, is related to substance dependence of human cancers and functions as a macrophage migration inhibitory factor^36^. The other enzyme encoded by a gene of interest, primary-amine oxidase, is expressed in mammals and involved in processes such as leukocyte trafficking and glucose metabolism^37^. These two enzymes might be possible therapeutic targets for cancer treatment. The other methods tended to not detect this kind of pathway because of the low ratio of metabolites and genes of interest involved. However, from the view of expression correlation, these nodes of interest in the phenylalanine metabolism subpathway had significantly higher average Pcc value than the entire corresponding pathway (Supplementary Table S1). Thus, Subpathway-CorSP effectively detected biologically meaningful pathways via emphasizing expression correlations between metabolites and genes of interest within metabolic pathways.

### Stability analysis and parameter analysis of Subpathway-CorSP

**Figure 5:**
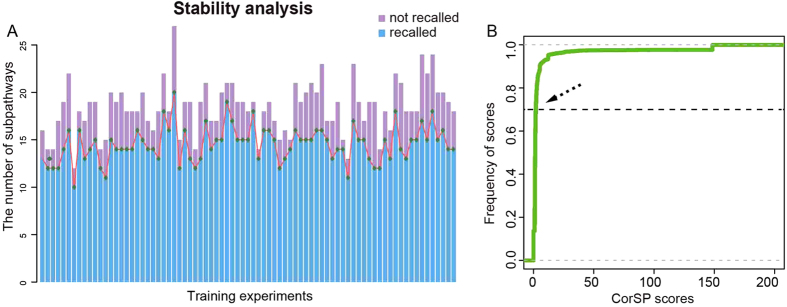
Stability analysis and parameter analysis of Subpathway-CorSP. (**A**) Histogram of stability analysis for Subpathway-CorSP. Horizontal axis, 80 training experiments; vertical axis, number of significant subpathways identified in corresponding training experiments. Blue, number of recalled original significant subpathways; purple, number of non-recalled original significant subpathways. (**B**) Cumulative distribution curve of CorSP scores. Arrow, score for 70% CorSP scores passing threshold m for all 150 metabolic pathways.

To validate the stability and reliability of the significant metabolic subpathways, stability analysis was performed. We extracted part of original samples as new data sets, and then identified new significant metabolic subpathways. We tested the number of original significant subpathways that were recalled. The more recalled subpathways, the more stable the subpathways that were identified. The steps were: First, keeping the metabolites and genes invariant, we simultaneously chose 75% of the benign prostate samples (Benign) and 75% of the prostate cancer samples (PCA+ Mets) without replacement from both the metabolomic profile and the matched gene expression profile. This yielded a training data set containing 12 benign prostate samples and 18 prostate cancer samples. Second, with the training data set, we performed Subpathway-CorSP strategy again and identified new significant metabolic subpathways. Third, the recalled number of original significant subpathways was counted, and the training experiment repeated 80 times. At least 12 and at most 18 significant subpathways were recalled (Fig. 5A). The recall rate was about 63.16–94.74%, and the average recall rate was up to 79.55%, although only 75% of the samples were used (Supplementary Table S2). Thus, we considered the significant metabolic subpathways identified by Subpathway-CorSP stable and reliable.

This study focused on identifying key metabolic subpathways by integrating expression correlations and topological features between nodes of interest within pathways. When two nodes of interest had higher Pcc value and smaller shortest path, they had larger CorSP score, which represented their close connection in a pathway. Thus, these two nodes tended to be added to one node set (that was a subpathway), and other nodes at their shortest path were added to the same node set simultaneously. Based on this theory, Subpathway-CorSP identified key subpathway regions in entire metabolic pathways. To set an appropriate threshold, m, for the identification of effective subpathways, we computed CorSP scores for each two nodes of interest in all metabolic pathways. We set m, by experience, to represent the score for 70% CorSP scores passing threshold m for all 150 metabolic pathways (Fig. 5B). The threshold m indicated the minimum permitted correlation extent at the shortest path between two nodes of interest in the pathway. A larger m value indicated that only nodes with higher expression correlations could be added to the same node set. The identified subpathways thus formed a smaller scale than a smaller m. Thus, we identified subpathways closely associated with metabolites and genes of interest. In contrast, decreasing m usually increased the number of other nodes except for nodes of interest within subpathways; it also increased the scale of the subpathways. Thus, we identified as many metabolites and genes associated with nodes of interest as possible.

### Subpathway-CorSP provided biologically informative models for metastatic prostate cancer

To evaluate the specific pathological features of metastatic prostate cancer and further demonstrate the use of Subpathway-CorSP, we identified metastatic prostate cancer-related metabolic subpathways. Considering different types of samples that were mixed into a single dataset, we used 12 localized prostate cancer samples (PCA) and 12 metastatic prostate cancer samples (Mets) to identify metabolites and genes of interest. Thus, 74 metabolites and 8765 genes of interest associated with metastatic prostate cancer were obtained (Table 1). Subpathway-CorSP analysis of this sub-group data set detected 103 potential subpathways from all metabolic pathways. Of these, 14 that corresponded to 14 entire pathways were identified at the 5% significance level (Supplementary Data set S1). These pathways were all associated with cancer in the literature (Table 3). Of these pathways, identified by Subpathway-CorSP, 13 were not identified by Pathway-G, 9 by Pathway-M, 8 by IMPaLA and 10 by Subpathway-GM; 5 pathways were simultaneously not detected by the four methods (Table 3).

**Figure 6:**
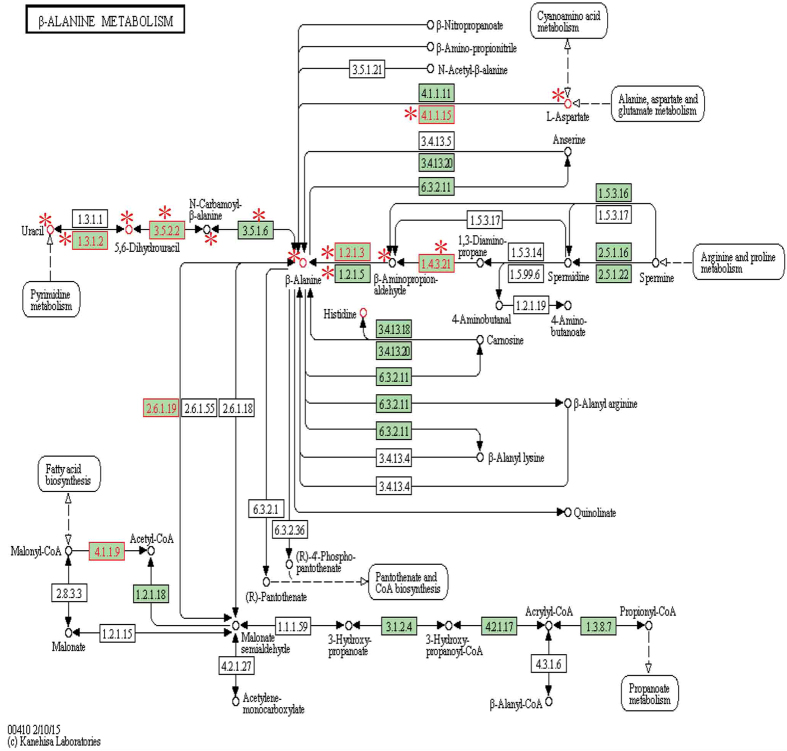
Beta-alanine metabolism pathway with metabolites and genes of interest annotated. Nodes near *symbol, key metabolism subpathway region (path:00410_2) identified by Subpathway-CorSP. The region contained key region of beta-alanine and uracil conversion. The molecules in this region formed a close-connected subpathway with high average Pcc value.

Special metabolic pathways only significantly associated with metastatic prostate cancer included purine metabolism (path:00230), the beta-alanine metabolism pathway (path:00410), pyrimidine metabolism (path:00240), the glycosphingolipid biosynthesis-globo series (path:00603) and the riboflavin metabolism pathway (path:00740). In these significant subpathways, nodes of interest showed denser expression correlations than the entire corresponding pathways. To the best of our knowledge, these pathways are not directly related to metastatic prostate cancer. However, the significant subpathway (path:00410_2) identified by Subpathway-CorSP captured a key region of beta-alanine and uracil conversion in the beta-alanine metabolism pathway (Fig. 6). Beta-alanine is a naturally occurring beta-amino acid found in vitamin B5 (pantothenic acid). Some research shows that the isomers of sarcosine, α-alanine and β-alanine, increase significantly as prostate cancer progresses to metastasis and are optimized as a group of potential prostate cancer biomarkers^38^. A metabolite of interest, uracil, is an effective and well tolerated regimen for hormone-refractory prostate cancer. Combinated with tegafur, uracil is effective in treating patients with prostate cancer^39^. The enzymes coded by genes of interest such as dihydropyrimidine dehydrogenase (NADP+) and dihydropyrimidinase, together with metabolites of interest such as beta-alanine, uracil and dihydrouracil, form a closely connected subpathway with high average Pcc value. Another significant drug metabolism subpathway (path:00983_4) may be also indirectly related to metastatic prostate cancer. In prostate cancer, chemotherapy drugs prompt the secretion of WNT16B by surrounding fibroblast cells, which activate cellular survival Wnt pathways in prostate cancer cells^40^. Metastasis of prostate cancer to bone increases morbidity. Several classes of drugs and treatments have been developed to interfere with oncogenes and oncoproteins known to be involved in the progression of prostate cancer into a more advanced form of disease^41^. Overall, these results suggested that specific metabolic subpathways that may be associated with metastatic prostate cancer were found by Subpathway-CorSP.

## Discussion

Metabolic pathways are important for interpreting metabolic functions of pathways related to specific diseases^16^,^17^,^18^. Integrative analysis of metabolites and genes based on pathway structure help to locate and evaluate key metabolic subpathways, and provide the opportunity to understand the mechanisms underlying cancer pathogenesis. From a biological perspective, dysfunctional genes are closely related to dysfunctional metabolites in pathways. Correlation analysis of them might be useful in helping to identify pathways^42^.

Research has been devoted to pathway analysis based on the idea of correlation. For example, EnrichNet performed a random walk with restart to identify crosstalk between known gene sets and pathway genes under the background of gene interaction networks^10^. The DECO algorithm is proposed to remove bias caused by correlation of expression data in gene-set analysis, and effectively improves the prediction accuracy of key pathways^11^. Wang *et al*. proposed an extension of the linear combination test, which is used for testing correlations between pathways and correlated multiple continuous phenotypes based on gene expression^12^. Using gene expression profiles, Guo *et al*. illustrated that separate analyses of up- and down- regulated genes identify more pathways^13^. These methods are effective for pathway analysis, but have limitations: First, they focused only on pathway genes, overlooking metabolites with vital functions in pathways. Second, they considered either network topology correlation or gene expression correlation, but not the two aspects simultaneously. Third, they predict pathways at the entire pathway level, but do not predict corresponding subpathway regions that may be more meaningful for reflecting relevant biological importance.

We developed a novel subpathway identification strategy called Subpathway-CorSP that integrated metabolites and genes of interest related to a given cancer into metabolic pathways. We identified key metabolic subpathway regions by systematically considering expression correlations and topological features between metabolites and genes of interest within the pathways. Especially, Subpathway-CorSP was an improvement on Subpathway-GM by emphasizing expression correlations between nodes of pathways. For the input data, Subpathway-GM treated DE genes and metabolites as interesting gene and metabolite sets. However, in Subpathway-CorSP, we integrated DE metabolites, DE genes, as well as metabolites and genes with high expression correlation related to the study condition as metabolites and genes of interest. Although the two methods were both based on expression profiles, Subpathway-CorSP further considered expression correlation information of metabolites and genes, defining metabolites and genes with high Pcc value as important. As a subpathway identification method, Subpathway-GM used a lenient distance similarity of signature nodes to locate subpathways. If the shortest path length between two signature nodes was shorter than n+1, then the two signature nodes and other non-signature nodes were added to the same node set. Thus, from a network-structure point of view, Subpathway-GM fully considered topological correlation between nodes within pathways. However, Subpathway-GM did not account for how metabolites and genes within the pathways correlated at the level of expression. Subpathway-CorSP further considered expression correlations of metabolites and genes based on topological features. When locating subpathways, we continued to apply lenient distance similarity and further improved it using CorSP score, instead of just the shortest path length between two nodes of interest. Specifically, for each pathway that contained nodes of interest, we computed Pcc and the shortest path between each two nodes of interest, and then computed CorSP score. When CorSP score between the two nodes of interest was greater than a given threshold, the nodes and other nodes at their shortest path were added to the same node set.

In this study, Subpathway-CorSP was mainly applied to prostate cancer data set. Many pathways corresponding to significant subpathways identified by Subpathway-CorSP were not detected by the traditional pathway identification methods Pathway-G, Pathway-M, IMPaLA and Subpathway-GM. The different results were mainly attributed to two points. One was the difference in use of the data set. Pathway-G used only DE genes, Pathway-M used only DE metabolites. The other difference was that Subpathway-CorSP considered not only pathway topology, but also expression correlations between metabolites and genes of interest. Both IMPaLA and Subpathway-GM did not use expression correlation information. Our analysis found that the average Pcc value for the 20 significant subpathways was significantly higher than for all the metabolic pathways. This result showed that significant subpathways identified by Subpathway-CorSP had clearly higher expression correlations. These results demonstrated that Subpathway-CorSP improved the identification of subpathways by considering expression correlations.

We focused on three of eight pathways identified by Subpathway-CorSP but not the other methods. We found they were highly associated with prostate cancer, suggesting that Subpathway-CorSP was able to identify more prostate cancer-related metabolic pathways. The stability analysis demonstrated stability and reliability of these significant metabolic subpathways. Flexibility could be introduced to this subpathway strategy by varying threshold m, which could be adjusted by users. As threshold m decreased, the number of the other nodes except for nodes of interest within subpathways increased, and thus the scale of the subpathways increased. Application of Subpathway-CorSP to a metastatic sub-group data set showed that Subpathway-CorSP located potential metastatic prostate cancer-related metabolic subpathways. The codes for Subpathway-CorSP can be downloaded at http://222.170.78.233/Subpathway-CorSP/.

We reported the implementation of a novel subpathway identification strategy to investigate the combined effect of metabolites and genes by integration of expression correlations and topological features within the metabolic pathways. The method had some limitations. Although KEGG is a popular and widely used pathway database resource, some true interactions might be missed when KEGG is used as the single data source^43^. To avoid biased results towards the single data source, in the future we plan to extend Subpathway-CorSP to a group of integrated pathway databases with topology structure, including KEGG^43^, WikiPathways^44^ and Reactome^45^. Based on multiple integrated pathway databases, more expression correlation information between nodes of the pathways could be obtained, which would further improve the effectiveness of Subpathway-CorSP for subpathway identification. In addition, some pathways, especially those from different pathway databases, partly overlap. Thus, correlations between nodes of pathways are possible. Although Subpathway-CorSP considered correlations arising from network topology, it did not consider correlations resulting from database design. This is a universal challenging situation in current pathway studies. A new strategy of reducing redundancy in pathways from integrated pathway databases might be needed. In addition, Subpathway-CorSP used hypergeometric test to evaluate pathway significance. This process assumed that pathways were independent of each other and did not consider correlations between nodes of pathways. When integrating multiple pathway databases, hypergeometric test would not be suitable to estimate the statistical significance of pathways. Thus, a new statistical method might need to be used in further research. Subpathway-CorSP has considerable potential for implementation of pathway identification associated with other molecules such as microRNAs or the long noncoding RNAs. This resource could help in studying interactions between these noncoding RNAs and human pathways and exploring crosstalk between pathways, greatly facilitating the understanding of the underlying mechanisms for complex diseases.

## Materials and Methods

### Data sets

Unbiased metabolomic profile including 16 benign prostate samples adjacent to tumor (Benign), 12 localized prostate cancer samples (PCA) and 12 metastatic prostate cancer samples (Mets) was from Sreekumar *et al*.^18^. With high-throughput liquid and gas chromatography-based mass spectrometry, high-throughput profiling of tissues quantitatively detected 626 metabolites, of which 518 were shared by the three diagnostic classes. These metabolites required a name-mapping step to standardize compound IDs^46^. MetaboAnalyst 3.0 (http://www.metaboanalyst.ca) is a comprehensive tool suite for metabolomic data analysis to convert metabolite names to KEGG compound IDs^47^. It has updated the underlying metabolite library based on the latest version of HMDB^48^. Especially, it has also reimplemented the algorithm to improve the performance of fuzzy string matching. Here, we used MetaboAnalyst 3.0 to perform name-mapping by entering 518 metabolite names. The database returned two kinds of results: query names in normal white indicated exact matches; query names highlighted in red indicated no exact or unique match, with multiple hits possible and users manually selecting the correct match if found (http://www.metaboanalyst.ca/ faces/Secure/utils/ConvertView.xhtml). We mapped the 518 metabolites to 100 metabolites with KEGG compound IDs. Matched gene expression profile data (16 Benign, 12 PCA and 12 Mets) was downloaded from the GEO database^49^. The accession number of gene expression profile was GSE8511 from platform GPL1708, containing 12,873 genes after processing.

## Methods

Subpathway-CorSP identified metabolic subpathway regions by integrating expression correlations and topological features between metabolites and genes of interest. Metabolic subpathways were identified mainly through: (i) mapping metabolites and genes of interest to graphs of metabolic pathways after graph-based reconstruction of metabolic pathways; (ii) developing subpathway identification algorithm and locating metabolic subpathways within pathways according to nodes of interest; (iii) evaluating the statistical significance of metabolic subpathway regions. The detailed steps are described below.

### Map metabolites and genes of interest to graphs of pathways

We constructed a bipartite network to represent metabolic pathways in the KEGG pathway database^50^,^51^. We downloaded KGML files (KEGG Markup Language, http://www.genome.jp/kegg/xml/) of KEGG pathways and converted them to list variables in R by applying iSubpathwayMiner, an R package developed by our research group^20^. We removed the map node in the corresponding KEGG pathway map and focused on molecules such as compounds and gene products. Resulting graphs mainly contained two types of nodes: compounds and enzymes. Edges were mainly constructed from reactions. Specially, if a compound participated in a reaction as a substrate or product, an undirected edge was used to connect the corresponding compound node to the enzyme node. Thus, substrates of a reaction were connected to enzyme nodes and the enzyme nodes were connected to products. Particularly, enzyme nodes used KEGG Orthology (KO) identifiers to overcome limitations of enzyme nomenclatures. KO identifiers integrated pathway and genome information, which have become a better controlled vocabulary for annotating genes to both metabolic and regulatory pathways^43^. In this way, positional information for metabolites and enzymes was extracted efficiently and used via a graph model that retained pathway structure.

Based on gene expression data and metabolomic experimental data, we respectively identified DE metabolites and DE genes using Wilcoxon rank-sum test and SAM method, and calculated Pcc for each metabolite-metabolite, metabolite-gene and gene-gene pair. We extracted metabolites and genes with Pcc greater than a cutoff such as 0.8, and regarded them as metabolites and genes with high expression correlation. We defined DE metabolites, DE genes, as well as metabolites and genes with high expression correlation as metabolites and genes of interest. Metabolites and genes of interest were mapped to corresponding nodes within pathway graphs. Notably, metabolites of interest were mapped to corresponding compound (substrate and product) nodes and genes of interest were assigned to KO identifiers and matched to enzyme nodes. Mapped nodes within each pathway graph were defined as nodes of interest.

### Locate subpathways according to nodes of interest

We developed a novel subpathway identification strategy to locate metabolic subpathway regions by systematically considering expression correlations and topological features between nodes of interest within given pathways. Specifically, for each pathway that contained nodes of interest, we computed Pcc and the shortest path between each two nodes of interest. A CorSP_ij_ score was computed to evaluate the expressional and topological influence between the ith and jth nodes of interest. CorSP_ij_ was defined as:


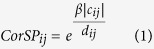


where, c_ij_ is Pcc between node i and node j based on their expression profiles, d_ij_ is the shortest path between node i and node j in any one pathway, calculated by breadth-first search algorithm, and β is a parameter set to balance the relationship between Pcc and the shortest path. In this study, we set β = 10.

We located subpathway regions according to CorSP scores between nodes of interest. There are many feasible distance measures in the area of computer science^52^,^53^. This study, we used the lenient distance similarity developed by our group^19^. We verified that the subpathways searched by lenient distance similarity were representative of the entire corresponding pathways for both topological centrality and biological interpretation^19^. We further improved lenient distance similarity using CorSP scores, instead of just the shortest path length between two nodes of interest. Specifically, if CorSP_ij_ between the ith and jth nodes of interest was greater than threshold m, the nodes and other nodes at their shortest path were added to the same node set. This process was recurrently computed for all nodes of interest. We extracted corresponding subgraphs in pathway graphs according to each node set, and defined these subgraphs with node number ≥s as subpathway regions because subgraphs with small scales could not usually form biologically significative subpathways.

### Evaluate statistical significance of metabolic subpathways

To evaluate the statistical significance of the located subpathways, hypergeometric tests were used, which required the following values: (i) number of metabolites and genes of interest submitted for analysis; (ii) number of background metabolites and genes; (iii) number of metabolites and genes of interest annotated to each metabolic subpathway; and (iv) number of background metabolites and genes annotated to each metabolic subpathway. All metabolites in the Human Metabolome Database (HMDB)^48^ and KEGG Human Pathway^43^ were used as background metabolites, and all human genes in KEGG as background genes. Then, the following formula was used to calculate P-value for the enrichment significance of the subpathways:


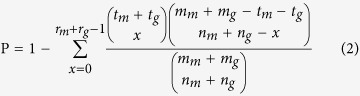


where, m_m_ (m_g_) was number of metabolites (genes) in the entire metabolome (genome), and n_m_ (n_g_) was number of metabolites (genes) of interest, of which r_m_ (r_g_) metabolites (genes) involved in the subpathway containing t_m_ metabolites (t_g_ genes).

## Additional Information

**How to cite this article**: Feng, C. *et al*. Subpathway-CorSP: Identification of metabolic subpathways via integrating expression correlations and topological features between metabolites and genes of interest within pathways. *Sci. Rep.* **6**, 33262; doi: 10.1038/srep33262 (2016).

## Supplementary Material

Supplementary Information

Supplementary Information

## Figures and Tables

**Table 1:** The number of metabolites and genes used in Subpathway-CorSP.

	DE Metabolites	DE Genes	Metabolites of Pcc > 0.8	Genes of Pcc > 0.8	Metabolites of interest	Genes of interest	Metabolites and Genes of interest
Benign vs. Cancer	48	1182	40	5581	63	5846	5909
PCA vs. Mets	44	1475	59	8738	74	8765	8839

**Table 2:** The significant subpathways identified by Subpathway-CorSP using prostate cancer data set (P < 0.05).

Subpathway ID	PathwayName	Subpathway-CorSP	Pathway-G	Pathway-M	IMPaLA	Subpathway-GM	Possible relation to the cancer	Reference (PMID)
path:00260_1	Glycine, serine and threonine metabolism	**0.00024269**	0.52409080	0.16216414	0.08498874	**0.00017964**	De novo Purine synthesis	19063642; 15229480
path:00563_1#	Glycosylphosphatidylinositol (GPI)-anchor biosynthesis	**0.00096023**	0.39629916	1	1	1	Suppress invasion of prostate and breast cancer cells	16822939
path:00510_4#	N-Glycan biosynthesis	**0.00137850**	0.11717815	1	1	1	Efficient detection of prostate cancer	18701493; 25154914
path:00562_1	Inositol phosphate metabolism	**0.00339828**	0.19556203	0.44168527	0.08637687	**0.00760810**	Membrane receptor signaling cascades and anti-cancer activity	14608114; 20414202
path:00340_1	Histidine metabolism	**0.00396946**	0.47151833	0.13647964	0.06435265	**0.00289440**	Cell proliferation	16203768; 18347416
path:00910_1	Nitrogen metabolism	**0.00434048**	0.72281060	0.0552906	**0.03996463**	**0.01352617**	Enhance anti-cancer activity	23746196; 26174441
path:00561_1#	Glycerolipid metabolism	**0.00464301**	0.93511527	0.38902790	0.36378593	0.29439641	High energy demand and cell propagation	18757836; 21802006
path:00601_1	Glycosphingolipid biosynthesis - lacto and neolacto series	**0.00573235**	0.16469429	1	1	**0.02863259**	Cell adhesion and the growth of tumor cells	20428086; 19658179
path:00052_1	Galactose metabolism	**0.00657521**	0.16469429	**0.02167594**	**0.00356990**	1	Toxic to ovarian cells	25586565; 21335998
path:00010_1	Glycolysis/Gluconeogenesis	**0.00947690**	0.46000521	0.37041772	0.17039408	**0.00059735**	Energy demand of colorectal cancer tissues	19678709; 19063642
path:00030_1	Pentose phosphate pathway	**0.00962547**	0.05589567	**0.01106147**	**0.00061828**	**0.00040717**	Increase cytotoxicity and oxidative stress	25560241; 24861463
path:00270_2#	Cysteine and methionine metabolism	**0.01530709**	0.12895515	1	1	1	Prostate cancer cell invasion and aggressivity	20718469; 21853037
path:00620_1	Pyruvate metabolism	**0.01530709**	0.64619119	1	1	**0.04290499**	Maintain of colon cancer cells	20919825; 18789002
path:00360_1#	Phenylalanine metabolism	**0.02129967**	1	0.23763160	1	1	High metabolic rate in cancer	20919825; 18789002
path:00330_1	Arginine and proline metabolism	**0.02749154**	0.95093432	**0.00182141**	**0.00173204**	**2.96E-05**	Colon cancer inhibition and tumoral energy production	20919825; 19678709
path:00983_4#	Drug metabolism-other enzymes	**0.02828285**	0.78112433	1	1	1	Metabolism of anti-cancer drug	21215737; 21533940
path:00450_1#	Selenoamino acid metabolism	**0.02872727**	0.16469429	1	1	0.07222314	As inhibitors of histonedeacetylase in human prostate cancer cells	19584079; 15041072
path:00670_1#	One carbon pool by folate	**0.02897254**	0.25510731	1	1	1		—
path:00770_1	Pantothenate and CoA biosynthesis	**0.03578242**	0.21421764	**0.00686790**	**0.00147122**	1		—
path:00480_1	Glutathione metabolism	**0.04742966**	0.93864190	**0.01768909**	**0.01660372**	**0.00389595**	Impact resistance of cancer cells	26056813; 25144624

Subpathways with # symbol are uniquely identified by Subpathway-CorSP.

**Table 3:** The significant subpathways identified by Subpathway-CorSP using metastatic prostate cancer data set (P < 0.05).

Subpathway ID	PathwayName	**Subpathway-CorSP**	Pathway-G	Pathway-M	IMPaLA	Subpathway-GM	Possible relation to the cancer	Reference (PMID)
path:00260_1	Glycine, serine and threonine metabolism	**3.88E-05**	0.64728481	**0.02756179**	**0.01784033**	1	De novo Purine synthesis	19063642; 15229480
path:00561_1#	Glycerolipid metabolism	**0.00037304**	0.42565344	0.36324046	0.15461455	0.12596684	High energy demend and cell propagation	18757836; 21802006
path:00563_1#	Glycosylphosphatidylinositol(GPI)-anchor biosynthesis	**0.00057710**	0.51188267	1	1	1	Suppress invasion of prostate and breast cancer cells	16822939
path:00910_1	Nitrogen metabolism	**0.00170052**	0.06603059	0.29898906	**0.01974242**	**0.02030707**	Enhance anti-cancer activity	23746196; 26174441
path:00230_1	Purine metabolism	**0.00253999**	0.12987834	**0.00018704**	**2.43E-05**	0.20697902	High DNA/RNA turnover in cancer	19063642
path:00562_1	Inositol phosphate metabolism	**0.00904761**	0.54447931	0.41370312	0.22525279	**0.02864760**	Membrane receptor signaling cascades and anti-cancer activity	14608114; 20414202
path:00410_2	beta-Alanine metabolism	**0.01083417**	0.44423116	**0.00070088**	**0.00031135**	1	Inhibit prostate cancer metastases	22824219; 21071389
path:00480_1	Glutathione metabolism	**0.01891404**	0.66681313	**0.01398493**	**0.00932534**	**0.01144082**	Impact resistance of cancer cells	26056813; 25144624
path:00510_3#	N-Glycan biosynthesis	**0.02567615**	0.22296249	1	1	1	Efficient detection of prostate cancer	18701493; 25154914
path:00983_4#	Drug metabolism-other enzymes	**0.02567615**	0.97396069	1	1	1	Metabolism of anti-cancer drug	21215737; 21533940
path:00240_2	Pyrimidine metabolism	**0.02716418**	0.22648719	**0.00770142**	**0.00174427**	1	Inhibition of metastatic prostate cancer	25360799; 25178642
path:00603_1	Glycosphingolipid biosynthesis- globo series	**0.02722552**	0.06424124	1	1	**0.00202817**	Tumor grade diagnosis and tumor prognosis	26132161; 25715344
path:00601_1#	Glycosphingolipid biosynthesis- lacto and neolacto series	**0.03676192**	1	1	1	1	Cell adhesion and the growth of tumor cells	20428086; 19658179
path:00740_2	Riboflavin metabolism	**0.04837857**	**0.03385502**	1	1	1	Increase metastatic ability of prostate cells	24791272; 17933458

Subpathways with # symbol are uniquely identified by Subpathway-CorSP.
