# Supplementary material for: Subpathway-CorSP: Identification of metabolic subpathways via integrating expression correlations and topological features between metabolites and genes of interest within pathways
Source: Sci Rep. 2016 Sep 14;6:33262. doi: 10.1038/srep33262 (PMC5021946; doi:10.1038/srep33262)
Supplement: Supplementary Information [file srep33262-s1.pdf]

Subpathway-CorSP: Identification of metabolic subpathways via integrating expression correlations and topological features between metabolites and genes of interest within pathways

Chenchen Feng[1,+], Jian Zhang[1,+], Xuecang Li[1,+], Bo Ai[1], Junwei Han[2], Qiuyu Wang[3], Taiming Wei[4], Yong Xu[5], Meng Li[1], Shang Li[2], Chao Song[6], Chunquan Li[1,*]

Supplementary Tables

Table S1. The average Pearson correlation coefficients of the 20 significant subpathways and the corresponding 20 entire pathways.

Subpathway ID	Average Pcc of the significant subpathway	Average Pcc of the entire corresponding pathway
path:00260_1	0.298506	0.219764
path:00563_1	0.298864	0.248137
path:00510_4	0.337163	0.229983
path:00562_1	0.277482	0.24809
path:00340_1	0.241407	0.20873
path:00910_1	0.222494	0.203142
path:00561_1	0.231522	0.228289
path:00601_1	0.272045	0.237186
path:00052_1	0.246715	0.223303
path:00010_1	0.251082	0.218861
path:00030_1	0.269308	0.228489
path:00270_2	0.262079	0.229685
path:00620_1	0.289397	0.236249
path:00360_1	0.32159	0.20644
path:00330_1	0.21834	0.198113
path:00983_4	0.257174	0.213068
path:00450_1	0.313966	0.23482
path:00670_1	0.235458	0.203566
path:00770_1	0.226276	0.213386
path:00480_1	0.238008	0.209706

Table S2. The results of stability analysis for the 20 significant subpathways identified by Subpathway-CorSP.

Training experiments	The number of new significant subpathways	The recalled number of original subpathways	The recall rate
experiment1	21	17	80.95%
experiment2	16	13	81.25%
experiment3	14	12	85.71%
experiment4	14	12	85.71%
experiment5	17	12	70.59%
experiment6	19	14	73.68%
experiment7	22	16	72.73%
experiment8	12	10	83.33%
experiment9	18	16	88.89%

experiment10	17	13	76.47%
experiment11	19	14	73.68%
experiment12	19	15	78.95%
experiment13	14	12	85.71%
experiment14	15	11	73.33%
experiment15	20	15	75.00%
experiment16	19	14	73.68%
experiment17	20	14	70.00%
experiment18	18	14	77.78%
experiment19	18	14	77.78%
experiment20	18	16	88.89%
experiment21	20	15	75.00%
experiment22	17	14	82.35%
experiment23	16	14	87.50%
experiment24	18	13	72.22%
experiment25	22	18	81.82%
experiment26	18	16	88.89%
experiment27	27	20	74.07%
experiment28	15	12	80.00%
experiment29	19	16	84.21%
experiment30	19	13	68.42%
experiment31	14	12	85.71%
experiment32	19	13	68.42%
experiment33	21	17	80.95%
experiment34	17	14	82.35%
experiment35	17	15	88.24%
experiment36	20	15	75.00%
experiment37	21	19	90.48%
experiment38	21	17	80.95%
experiment39	19	15	78.95%
experiment40	19	15	78.95%
experiment41	18	15	83.33%
experiment42	19	18	94.74%
experiment43	14	13	92.86%
experiment44	18	16	88.89%
experiment45	19	16	84.21%
experiment46	17	15	88.24%
experiment47	15	12	80.00%
experiment48	16	13	81.25%
experiment49	15	14	93.33%
experiment50	21	16	76.19%
experiment51	19	15	78.95%
experiment52	20	15	75.00%
experiment53	21	15	71.43%
experiment54	20	16	80.00%
experiment55	23	16	69.57%
experiment56	17	15	88.24%
experiment57	17	13	76.47%
experiment58	19	14	73.68%

experiment59	15	14	93.33%
experiment60	13	11	84.62%
experiment61	23	17	73.91%
experiment62	19	15	78.95%
experiment63	17	15	88.24%
experiment64	19	13	68.42%
experiment65	19	12	63.16%
experiment66	14	12	85.71%
experiment67	18	15	83.33%
experiment68	16	13	81.25%
experiment69	22	18	81.82%
experiment70	21	14	66.67%
experiment71	18	13	72.22%
experiment72	18	15	83.33%
experiment73	18	15	83.33%
experiment74	24	17	70.83%
experiment75	22	15	68.18%
experiment76	24	18	75.00%
experiment77	20	15	75.00%
experiment78	20	16	80.00%
experiment79	19	14	73.68%
experiment80	18	14	77.78%

Supplementary Figures

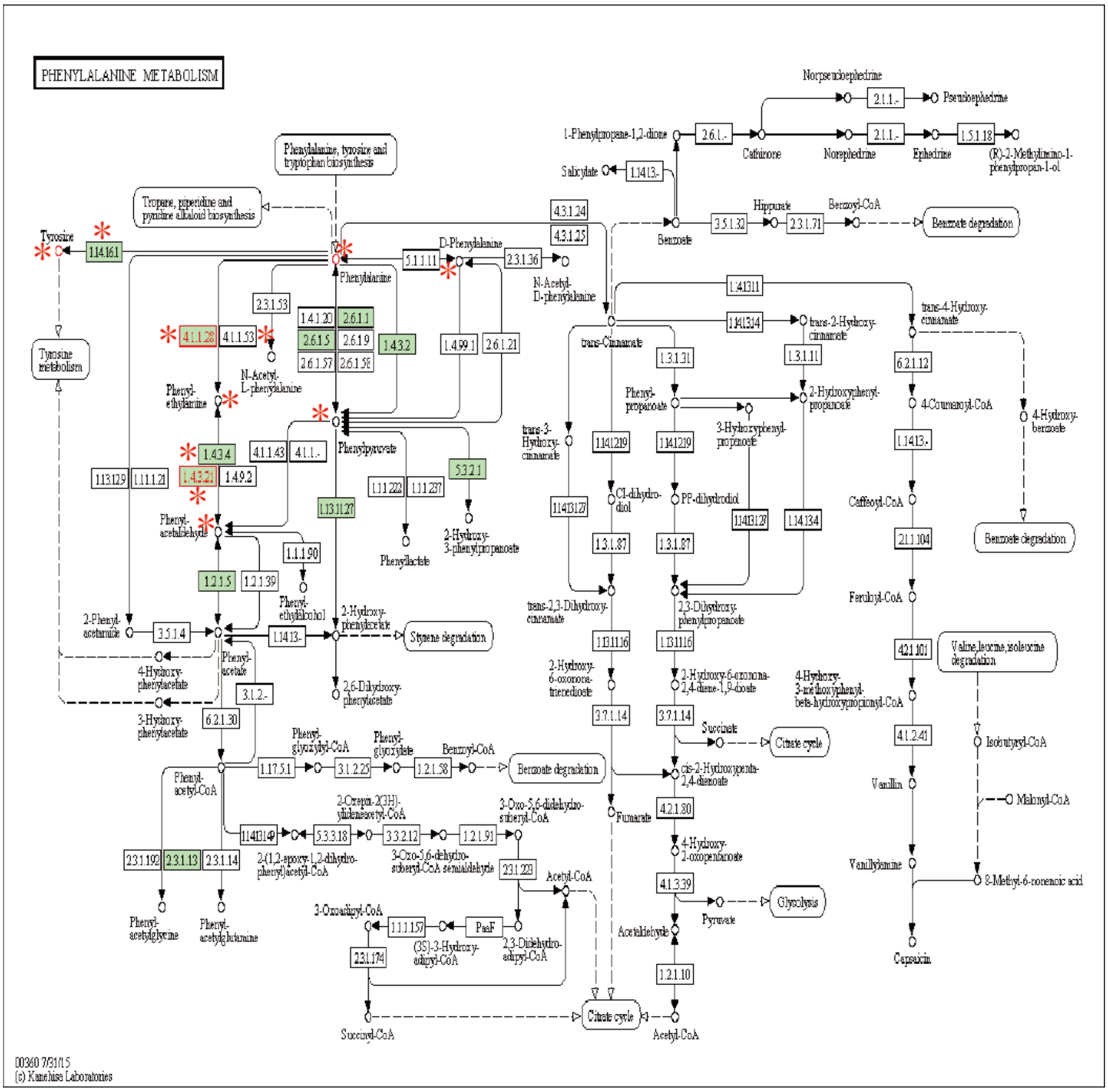

Figure S1 Phenylalanine metabolism pathway with metabolites and genes of interest annotated.

Nodes near * symbol, key metabolism subpathway region (path:00360_1) identified by Subpathway-CorSP.

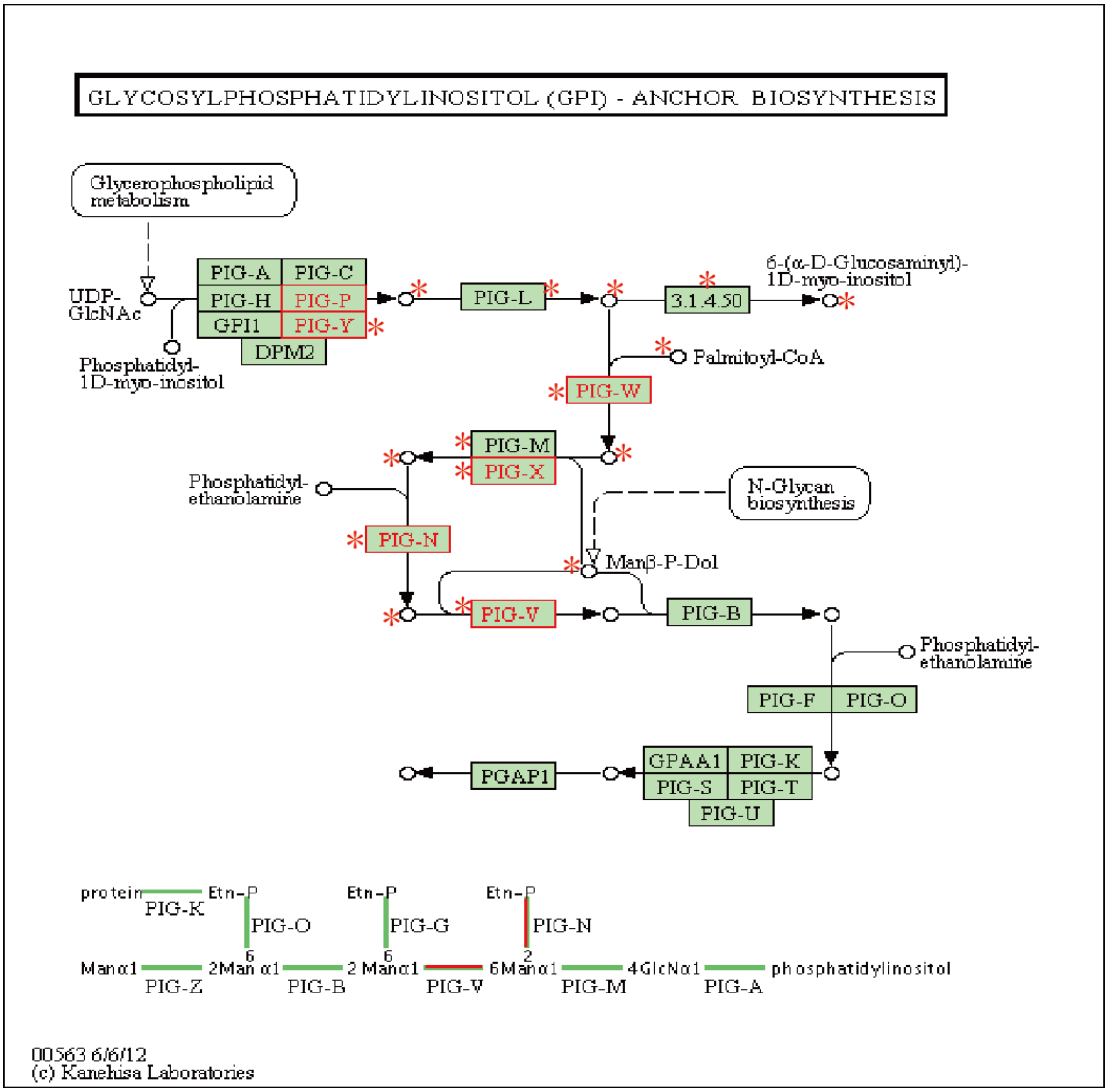

Figure S2 Glycosylphosphatidylinositol (GPI)-anchor biosynthesis metabolism pathway with metabolites and genes of interest annotated.

Nodes near * symbol, key metabolism subpathway region (path:00563_1) identified by Subpathway-CorSP.

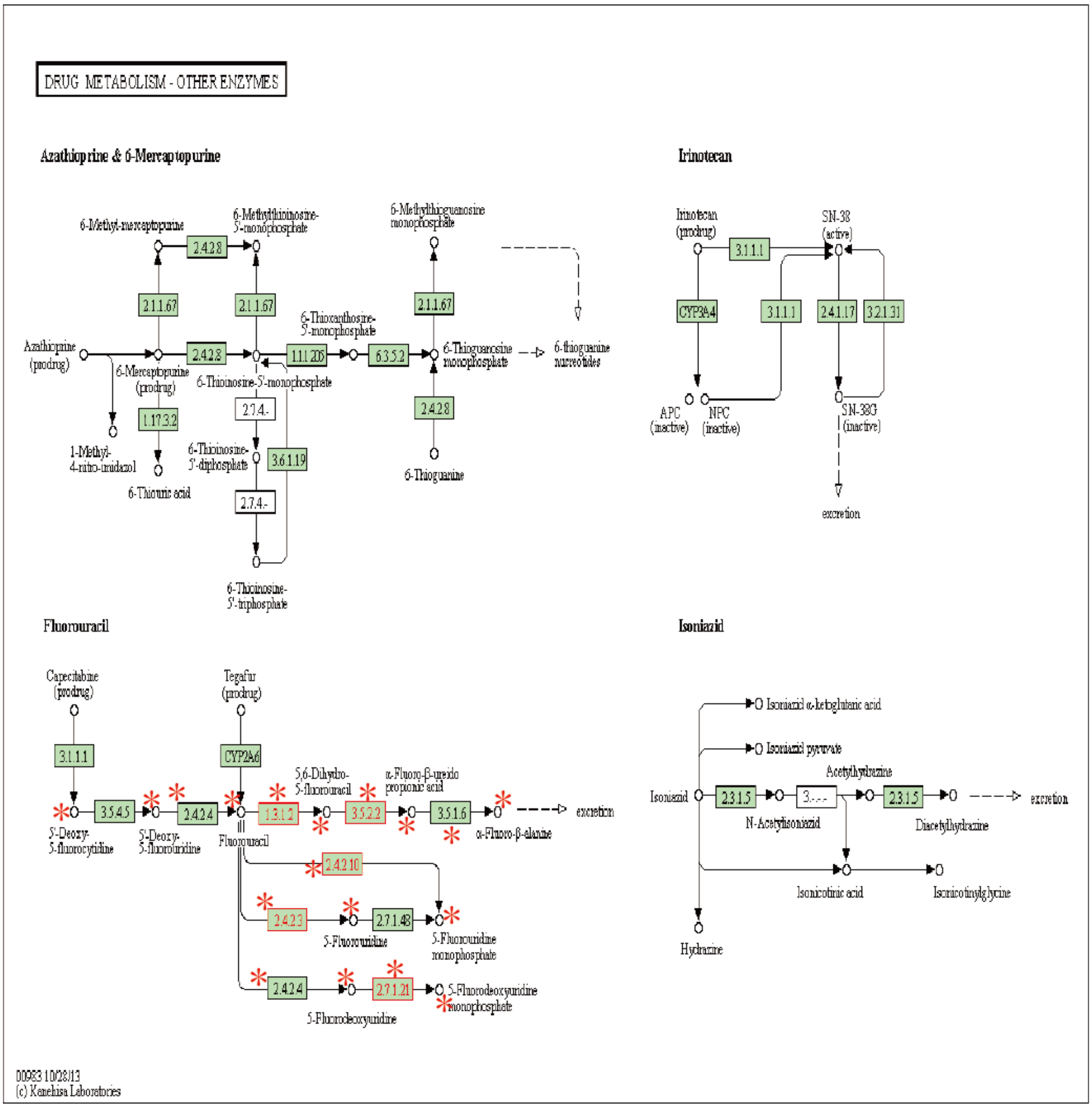

Figure S3 Drug metabolism-other enzymes metabolism pathway with metabolites and genes of interest annotated.

Nodes near * symbol, key metabolism subpathway region (path:00983_4) identified by Subpathway-CorSP.

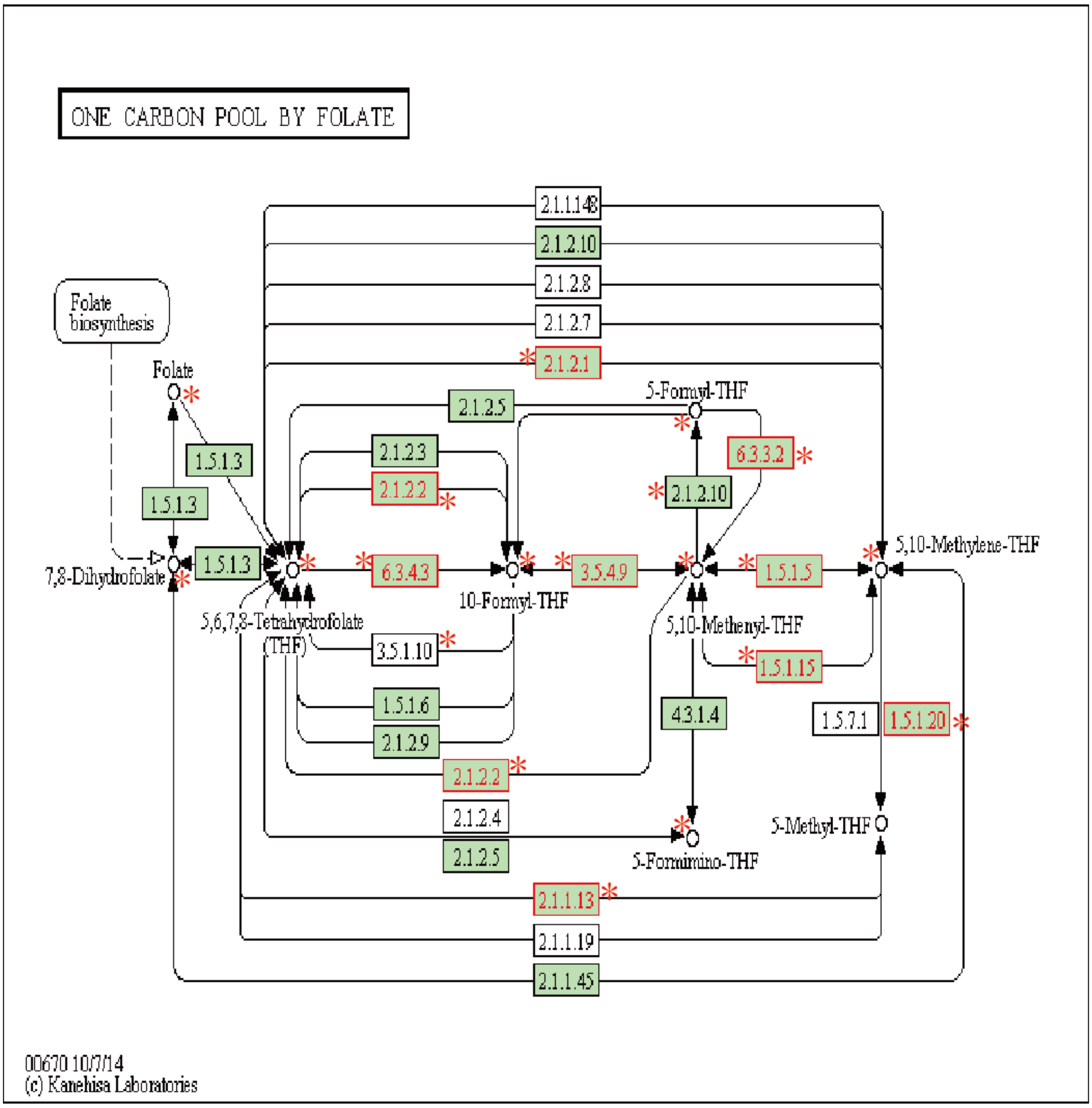

Figure S4 One carbon pool by folate metabolism pathway with metabolites and genes of interest annotated.

Nodes near * symbol, key metabolism subpathway region (path:00670_1) identified by Subpathway-CorSP.
